# Fine-Tuning Homology-Directed Repair (HDR) for Precision Genome Editing: Current Strategies and Future Directions

**DOI:** 10.3390/ijms26094067

**Published:** 2025-04-25

**Authors:** Sibtain Haider, Claudio Mussolino

**Affiliations:** 1 Institute for Transfusion Medicine and Gene Therapy, Medical Center—University of Freiburg, 79106 Freiburg, Germany; sibtain.haider@uniklinik-freiburg.de; 2 Center for Chronic Immunodeficiency (CCI), Medical Center—University of Freiburg, 79106 Freiburg, Germany; 3 Faculty of Medicine, University of Freiburg, 79106 Freiburg, Germany

**Keywords:** CRISPRCas9, HDR, NHEJ, DNA repair

## Abstract

CRISPR–Cas9 is a powerful genome-editing technology that can precisely target and cleave DNA to induce double-strand breaks (DSBs) at almost any genomic locus. While this versatility holds tremendous therapeutic potential, the predominant cellular pathway for DSB repair—non-homologous end-joining (NHEJ)—often introduces small insertions or deletions that disrupt the target site. In contrast, homology-directed repair (HDR) utilizes exogenous donor templates to enable precise gene modifications, including targeted insertions, deletions, and substitutions. However, HDR remains relatively inefficient compared to NHEJ, especially in postmitotic cells where cell cycle constraints further limit HDR. To address this challenge, numerous methodologies have been explored, ranging from inhibiting key NHEJ factors and optimizing donor templates to synchronizing cells in HDR-permissive phases and engineering HDR-enhancing fusion proteins. These strategies collectively aim to boost HDR efficiency and expand the clinical and research utility of CRISPR–Cas9. In this review, we discuss recent advances in manipulating the balance between NHEJ and HDR, examine the trade-offs and practical considerations of these approaches, and highlight promising directions for achieving high-fidelity genome editing in diverse cell types.

## 1. Introduction

Genome editing technologies have revolutionized our ability to manipulate genetic material with unprecedented precision, thereby opening new avenues for both fundamental research and therapeutic innovation. Among the most impactful advances in this field is the development of programmable nucleases—namely, zinc-finger nucleases (ZFNs), transcription activator-like effector nucleases (TALENs), and the CRISPR–Cas9 system [1,2,3]. These tools enable targeted modifications at nearly any genomic locus. Although ZFNs and TALENs laid the groundwork for site-specific genome editing, their intricate design requirements have limited widespread application. In contrast, the RNA-guided mechanism of CRISPR–Cas9 has streamlined target recognition and spurred rapid adoption across diverse applications, ranging from disease modelling to gene therapy [4].

A critical factor influencing editing outcomes is the manner in which cells repair the double-strand breaks (DSBs) induced by these nucleases. In mammalian cells, the predominant repair pathways are non-homologous end-joining (NHEJ) and homology-directed repair (HDR). NHEJ operates quickly, often yielding small insertions or deletions (indels), whereas HDR employs a homologous donor sequence to achieve precise genetic modifications [5,6,7]. Other mechanisms, such as microhomology-mediated end-joining (MMEJ) and single-strand annealing (SSA), also contribute to DSB repair, typically resulting in similar or larger deletions [8,9]. Although newer CRISPR-based approaches—such as base editing and prime editing—seek to minimize indels by leveraging nickases coupled with deaminases or reverse transcriptase, these methods are generally optimized for small-scale modifications [10,11]. For larger or more complex genetic alterations, the HDR-mediated repair of nuclease-induced DSBs remains indispensable, particularly when the precise correction of diverse disease-causing mutations is required.

The choice between these repair pathways is not merely a biochemical curiosity but has profound implications for therapeutic applications. For instance, while NHEJ is highly efficient and often exploited for gene disruption strategies, its error-prone nature limits its utility for applications requiring precision [12]. Conversely, HDR, though less efficient under many conditions, is the preferred route for achieving high-fidelity genome modifications. Consequently, a major focus in genome engineering is the development of strategies to bias repair events toward HDR while suppressing the faster but error-prone NHEJ pathway. Extensive efforts to elucidate the molecular details of DSB repair have not only identified actionable targets for modulating these pathways but have also revealed potential pitfalls such as off-target effects and genomic instability, which continue to challenge the field [5,6,13].

## 2. DNA Repair Pathways

While DNA repair pathways have been described in detail previously in the literature, we provide only a brief summary of the relevant DNA repair mechanisms [14,15], focusing on emerging strategies to enhance HDR-mediated repair and ultimately improve the reliability and precision of genome editing.

### 2.1. Non-Homologous End-Joining (NHEJ)

**Figure 1:**
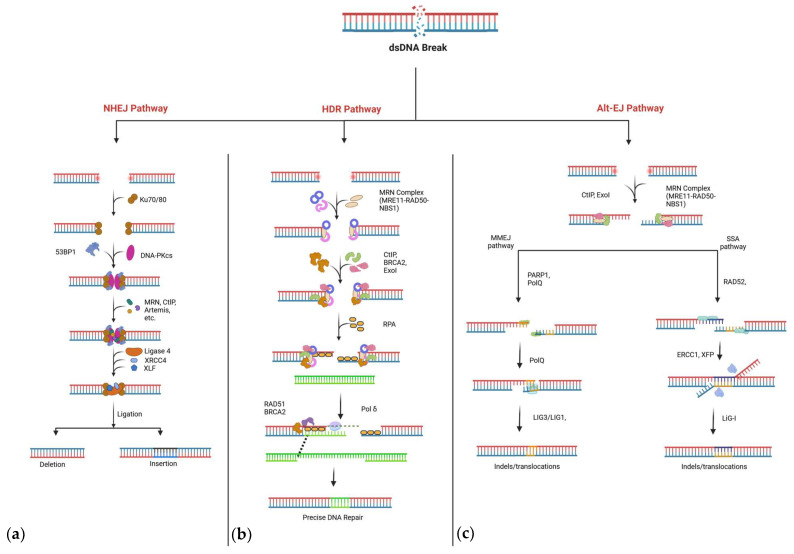
Overview of major DNA double-strand break (DSB) repair pathways. (**a**) NHEJ: Ku70/Ku80 binds broken DNA ends and recruits DNA-PK_cs; damaged ends are trimmed (often by Artemis), and the XRCC4–Ligase IV complex seals the break. (**b**) HDR: The MRN complex initiates resection, generating 3′ overhangs coated by RPA. RAD51, aided by BRCA2 and PALB2, replaces RPA and mediates strand invasion into a homologous template, followed by DNA synthesis (Pol δ) and ligation. (**c**) Alt-EJ: Includes two error-prone sub-pathways. MMEJ relies on Pol θ (regulated by PARP1) and short microhomologies. SSA involves extensive resection and direct annealing of homologous repeats by RAD52, resulting in deletion of intervening sequences. Figure is created with BioRender.com.

NHEJ is frequently described as the cell’s “first responder” to DSBs and proceeds with minimal end resection. In the canonical version of NHEJ (cNHEJ), the Ku70–Ku80 heterodimer first recognizes and binds to broken DNA ends, effectively preventing extensive resection [7,16,17] (Figure 1a). Simultaneously, p53-binding protein 1 (53BP1) may be recruited to reinforce the protection of the DNA ends and inhibit BRCA1, a key HDR factor. This early binding event locks the break site into an NHEJ-favored repair state [18,19]. After Ku70–Ku80 binding, DNA-dependent protein kinase catalytic subunit (DNA-PKcs) accumulates at the break, helping align the damaged ends and orchestrating the recruitment of nucleases or polymerases to modify them if necessary [13]. In many cases, Artemis endonuclease removes overhanging nucleotides, while Pol μ or Pol λ may fill in small gaps [20,21,22]. Once both ends are suitably “cleaned” or “processed”, XRCC4 and DNA ligase IV perform the final ligation step [23,24].

In principle, NHEJ can be remarkably accurate when the broken ends are blunt or nearly so. However, the CRISPR-Cas9 nuclease generates DSBs that are usually re-cleaved if the protospacer adjacent motif (PAM) or guide RNA (gRNA) target sequence is not disrupted. The continual re-cutting of the locus favors small insertions or deletions at the break site. Moreover, the asymmetry of Cas9 cutting, where one DNA strand is cleaved prior to the other, can create a temporal window in which one end is processed while the other remains bound by Cas9, potentially shifting the balance toward indel formation [25]. NHEJ remains active throughout all cell cycle phases, making it a rapid and highly utilized repair mechanism in both proliferating and quiescent cells. However, its tendency to produce disruptive mutations in the presence of a designer nuclease makes it unsuitable for most precise gene-editing applications [26]. Therefore, many current genome-engineering approaches seek to reduce NHEJ efficiency while simultaneously enhancing HDR as discussed below.

### 2.2. Homology-Directed Repair (HDR)

HDR provides a high-fidelity alternative to NHEJ by harnessing homologous donor templates—often sister chromatids in S/G2 phases to direct error-free repair [27]. In the earliest steps of HDR, the MRN complex (MRE11–RAD50–NBS1) identifies the break and partially resects the 5′ ends with CtIP, leading to short 3′ single-stranded overhangs [28,29] (Figure 1b). Long-range resection by Exo1 and the Dna2/BLM helicase complex then generates extended 3′ ssDNA tails, which are protected by replication protein A (RPA) [30]. RPA helps prevent secondary structure formation, after which RAD51 displaces RPA and forms nucleoprotein filaments that perform a homology search.

Upon locating a suitable donor sequence, often located on the sister chromatid, the RAD51-ssDNA filaments initiate strand invasion to form a displacement loop (D-loop) [31]. DNA polymerase extends the invading strand using the homologous sequence as a template, and the repair can proceed via two main routes. In the double-strand break repair (DSBR) sub-pathway, two Holliday junctions may form and be resolved into either crossover or non-crossover products. Alternatively, synthesis-dependent strand annealing (SDSA) involves the dissociation of the newly extended strand from the donor and annealing to the complementary 3′ end on the opposite side of the break, yielding exclusively non-crossover outcomes [32,33,34]. Although HDR is highly accurate, its reliance on both extensive resection and a homologous donor template typically confines it to S/G2 phase cells [27]. This cell-cycle restriction, combined with the complex orchestration of resection, strand invasion, and synthesis, makes HDR a less frequent pathway than NHEJ in many cell contexts.

### 2.3. Alternative Repair Pathways: MMEJ and SSA

Although NHEJ and HDR dominate discussions of CRISPR repair outcomes, alternative pathways like MMEJ (also referred to as polymerase theta-mediated end-joining, or TMEJ) and SSA can also contribute to Cas9-induced DSB repair [21] (Figure 1c). Both involve end resection to expose short or long regions of homology. In MMEJ, microhomologies ranging from two to twenty nucleotides guide the annealing of opposing DNA ends. DNA polymerase theta (Pol θ), assisted by poly (ADP-ribose) polymerase 1 (PARP1), typically mediates this process [35,36,37]. Because MMEJ can delete sequences between these micro-homologous regions, it often generates moderate-to-large deletions and is considered highly error-prone [9,38].

SSA requires more extensive stretches of homology (usually >20 nucleotides) flanking the DSB. Exo1, DNA2, or BLM helicase resects the ends, exposing these homologous sequences, which then anneal under the influence of RAD52. As in MMEJ, the intervening DNA is excised, causing large deletions [39,40,41]. Notably, both MMEJ and SSA require resection and hence are more likely to occur when NHEJ is suppressed or when the break persists in the S/G2 phase. While these alternative pathways can rescue cell viability if canonical pathways fail, their propensity to cause deletions often undermines precise genome-editing goals.

#### Competition Between DNA Repair Pathways

While all DNA repair pathways contribute to maintaining genome integrity, multiple pathways can be activated in response to a DSB caused by Cas9. In such cases, these pathways often compete with one another to repair the damage, but the outcome fundamentally depends on whether DNA ends become resected, a process facilitated by MRN, CtIP, and other factors, and whether a homologous donor is available [28]. Moreover, cell-cycle status plays a pivotal role, as HDR machinery is most active in the S/G2 phases while NHEJ operates throughout all phases. Proteins such as 53BP1 and the Shieldin complex stabilize DNA ends against resection, favoring NHEJ, whereas BRCA1 and CtIP promote resection and HDR. At the practical level, CRISPR-Cas9 gene editing frequently yields predominantly NHEJ-driven indels, with HDR events constituting only a minority of repair outcomes. Therefore, boosting HDR has become a major research focus and different approaches have been utilized to achieve this goal.

## 3. Strategies to Enhance HDR

**Figure 2:**
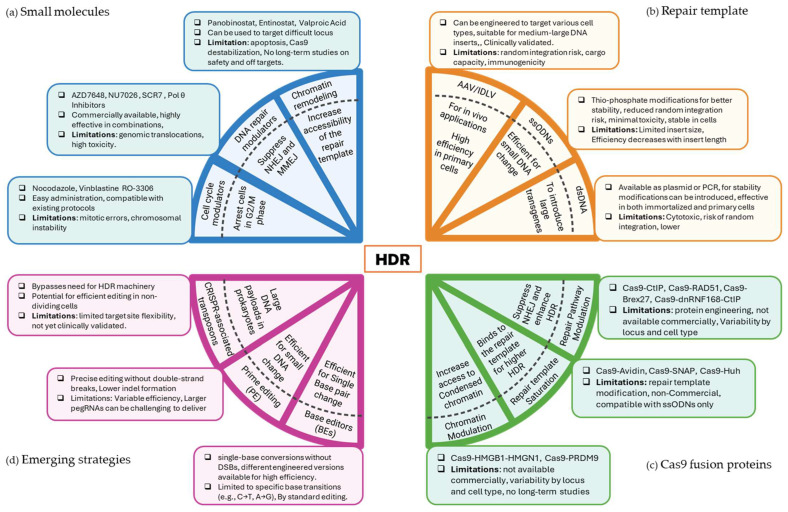
Schematic overview of strategies used to enhance HDR efficiency in CRISPR-based genome editing. The figure is divided into four quadrants, each representing a broad category of approaches: (**a**) Small molecules (blue): This section highlights the use of cell cycle modulators, DNA repair inhibitors, and chromatin remodelers to shift cells into HDR-permissive phases and increase template accessibility. (**b**) Repair templates (orange): This section compares different DNA donor formats—AAV for efficient in vivo and primary cell delivery, ssODNs for small changes, and dsDNA for larger inserts. (**c**) Cas9 Fusion proteins (green): This section depicts the fusion of Cas9 to HDR-promoting factors, chromatin modifiers, and repair pathway modulators that suppress competing end-joining pathways and enhance template-driven repair. (**d**) Emerging strategies (pink): This section showcases newer innovations such as base editors for single base-pair changes, prime editing for precise sequence alterations without double-strand breaks, and CRISPR-associated transposons for large payloads. Figure is created with BioRender.com.

An early approach includes transiently suppressing NHEJ factors (e.g., 53BP1, DNA-PKcs, or Ku70/Ku80) via small-molecule inhibitors, RNA interference, or CRISPR-based knockdown. Other strategies involve synchronizing cells in S/G2 phases, overexpressing or fusing HDR-promoting proteins (such as RAD51, CtIP, or BRCA2) directly to Cas9, and optimizing donor constructs so that the repair machinery can find and integrate them more efficiently (Figure 2). We will further discuss these strategies in more detail and the shortcomings associated with them with regards to their use for clinical therapy.

### 3.1. Enhancing HDR Efficiency with Small Molecules

One of earliest approaches developed was the use of small molecules to enhance HDR by modulating cell cycle progression, inhibiting competing repair pathways, or improving chromatin accessibility (Table 1). Cell cycle modulators, such as nocodazole, vinblastine, and RO-3306, facilitate HDR by arresting cells in the G2/M phase, a period conducive to homologous recombination [42,43]. Similarly, XL413, a CDC7 inhibitor, extends S phase duration, allowing more time for HDR [44]. Another effective approach involves inhibiting NHEJ and MMEJ, which compete with HDR. For example, DNA-PK inhibitors (e.g., AZD7648 and NU7026) and ligase IV inhibitors (e.g., SCR7) suppress NHEJ [45,46,47,48], while polymerase θ inhibitors (e.g., ART558, PolQi1, and PolQi2) target MMEJ. ART558 selectively inhibits Polθ polymerase activity, reducing TMEJ-mediated DNA repair and demonstrating synthetic lethality in HR-deficient cells, such as those with BRCA1/2 mutations [49,50]. Additionally, PolQi1 and PolQi2 have been identified as potent inhibitors of Polθ activity in the dual-inhibition strategy known as 2iHDR. This approach reduces mutagenic events, such as large deletions and translocations, while improving knock-in outcomes across various cell types and loci [51]. Small molecules like RS-1 and farrerol enhance RAD51 activity or recruitment to double-strand breaks, directly promoting HDR [52].

Chromatin remodeling agents further optimize HDR by increasing the accessibility of the donor template. Histone deacetylase (HDAC) inhibitors, such as panobinostat and entinostat [53], relax chromatin, while ATR inhibitors (VE-822) and CHEK1 inhibitors (AZD-7762) regulate DNA damage response pathways to favor HDR [54]. Emerging studies on valproic acid suggest it facilitates chromatin refolding, though its thermal destabilization of Cas9 raises concerns [55,56]. Despite their potential, HDR-enhancing small molecules often introduce challenges, including cytotoxicity and genomic instability. Therefore, a thorough understanding of their cellular effects and impact on genome editing outcomes is essential for their effective and safe application.

**Table 1:** Small molecules targeting DNA repair pathways to enhance HDR.

Target Pathway or Mechanism	Small Molecules	Cell Lines	HDR Increase	Adverse Effects
Cell Cycle Modulation	Nocodazole [43]	HEK293T, HDFn, and H9 ESCs	Up to 3-fold	Mitotic errors and chromosomal instability [57]
	Vinblastine [42]	HEK293T, HeLa, and HepG2	Up to 6-fold	
	RO-3306 and XL413 [44]	K562 and T cells	Up to ~5-fold	
NHEJ Inhibition	AZD7648 [45]	HSPCs and fibroblasts	>90% HDR	Genomic instability and inconsistent effectiveness [58,59]
	NU7026 [46,47]	HEK293, K562, and hiPSCs	Between ~3- and ~7-fold	
	SCR7 [48]	MelJuSo, HEK293T, and DC2.4	Up to 19-fold	
	DP308 [60]			
MMEJ Suppression	Pol θ inhibitors ART558 [49]	mESC, HEK293T, HeLa, U2OS, and RPE1	Up to 2-fold	Genomic instability and lethality in HR-deficient cells [50]
	PolQi1 and PolQi2 [51]	HEK293T, Jurkat HepG2, hiPSCs, T cells, and PHH	~80% HDR	
RAD51 Activation	RS-1 [52]	HEK-293 and U2OS	Between ~3- and ~6-fold	Limited validation; long-term effects unknown
	Farrerol [61]	HEK 293FT, mESCs, and HCA2-hTERT	Up to ~3-fold	
RPA-ssDNA Interaction	NSC15520 [46]	HEK293, K562, and h_iPSCs	Between ~3- and ~7-fold	Limited studies
Chromatin Remodeling	Panobinostat and entinostat [53]	H27, HEK293t, and HeLa	Up to ~3-fold	Cytotoxicity and apoptosis [53]
	Valproic acid [55]	mESCs	Significant	Cas9 destabilization (valproic acid) [56]
DNA Damage Response	VE-822 and AZD-77 [54]	hPSCs	Up to 6-fold	Limited studies; potential off-target effects [54]

HDR increases listed are based on studies employing small molecule modulators targeting DNA repair pathways, cell cycle regulation, or chromatin dynamics. However, many compounds pose risks of chromosomal instability, apoptosis, or inconsistent efficacy. Reported fold increases and effects are highly cell-type and locus dependent, and long-term safety data is limited for several compounds.

#### Adverse Effects of HDR-Enhancing Small Molecules

DNA-PK inhibitors like AZD7648, while suppressing NHEJ, can induce kilobase-scale deletions, chromosome arm loss, and translocations, leading to significant genomic instability [58]. Similarly, NHEJ inhibitors like SCR7 show inconsistent efficacy, limiting their application across cell types [59]. Cell cycle modulators, including nocodazole [57,62] and ABT-751 [63], may lead to chromosomal instability and mitotic errors. HDAC inhibitors, though effective in chromatin remodeling, are associated with cytotoxicity and apoptosis at higher doses [53,54]. Polθ inhibitors, such as ART558, PolQi1, and PolQi2, selectively target TMEJ but can induce synthetic lethality in cells with BRCA mutations or Shieldin complex defects [50]. Moreover, prolonged Cas9 activity induced by some small molecules increases risks of off-target effects, immune responses, and genotoxicity [64]. Variability in drug performance across cell types adds another layer of complexity, emphasizing the need for optimized dosing and delivery systems to ensure safety and efficacy in clinical applications. Moreover, the DNA repair pathway utilized by the cell is strongly influenced by the type of repair template and the nature of the intended edit. Therefore, selecting an appropriate optimized repair template is also critical to achieve successful HDR events.

### 3.2. Optimizing Repair Templates to Enhance HDR

Several types of repair templates, including viral vectors, single-stranded DNA (ssDNA) templates, and double-stranded DNA (dsDNA), have been employed, each offering distinct advantages and limitations (Table 2). Below, we discuss these approaches in detail, highlighting their modifications, efficiencies, and challenges.

#### 3.2.1. Viral-Based Repair Templates

Viral vectors, such as recombinant adeno-associated viruses (rAAVs), are widely used as HDR templates due to their ability to deliver single-stranded DNA with high efficiency [65]. rAAV templates exhibit robust HDR outcomes, particularly in hard-to-edit primary cells and in vivo applications [66]. Capsid engineering, such as the development of Y704T variants, has further enhanced delivery specificity and reduced immunogenicity [67]. Moreover, modifications to the surface of viral capsids, such as conjugation with HDR-promoting proteins, have shown potential to improve editing efficiency [68]. Advancements in rAAV-based systems include synthetic RNA-targeting sequences embedded in the repair template, allowing precise linearization and reducing random integration events [69]. Despite these improvements, the use of viral-based templates poses challenges, including low-frequency integration events, AAV’s limited packaging capacity (~4.7 kb), risks of insertional mutagenesis, and immune responses against viral components [70,71]. These limitations emphasize the need for virus-free genome editing strategies and alternative delivery systems.

Integrase-defective lentiviral vectors (IDLVs) have also emerged as a valuable platform for delivering donor templates in HDR-based genome editing [72]. Their ability to accommodate medium-to-large DNA sequences (up to ~9 kb) and transduce both dividing and non-dividing cells makes them particularly advantageous in therapeutic settings. Unlike integrating lentiviral vectors, IDLVs support transient expression without stable genomic integration, thereby reducing the risk of insertional mutagenesis. IDLVs have been effectively used alongside engineered nucleases to facilitate targeted gene correction in various cell types, including human stem and progenitor cells [73]. Notably, studies have reported up to 50% gene knock-in efficiency in human ESCs and the efficient correction of disease genes such as *IL2RG*, *ADA*, and *CD40LG* in clinically relevant models [74,75,76]. Compared to AAVs, IDLVs induce a milder DNA damage response and show lower frequencies of unwanted vector DNA insertions, improving the genomic safety profile in hematopoietic stem cells [77,78]. One of the main challenges is lower transduction efficiency in certain primary cells which requires further engineering and is a subject of continuous interest. Moreover, double-IDLV systems have been developed to expand their cargo capacity [76]. These advancements underscore the growing utility of IDLVs as an efficient and safer alternative for HDR-mediated genome editing.

#### 3.2.2. Single-Stranded DNA (ssDNA) Templates

Single-stranded oligonucleotides (ssODNs) are a highly versatile and efficient repair template for HDR, particularly for introducing small modifications such as point mutations or single-nucleotide polymorphisms. The length and symmetry of homology arms in ssODNs significantly impacts HDR efficiency, with arms of 60–80 nucleotides often yielding optimal results [79,80,81]. Chemical modifications, such as phosphorothioate linkages, enhance template stability and resistance to nucleases [82,83]. Innovative approaches have emerged to improve ssODN-based HDR. For example, modular ssDNA donors designed with RAD51-binding sequences have shown an increased recruitment of the homologous recombination machinery, enhancing HDR precision [84]. Retron systems, which generate ssODNs intracellularly, offer another promising strategy by providing high concentrations of templates without extracellular degradation [85,86]. Furthermore, covalent linking of ssODNs to Cas9 RNPs or nanoparticles facilitates nuclear delivery, resulting in a significant improvement in editing efficiency across multiple cell types [87,88,89]. Recent advancements also include transcription-coupled donor DNA expression systems, which provide a continuous supply of ssDNA templates in cells. These systems, leveraging transcriptional machinery, enhance HDR efficiency by synchronizing template availability with DNA repair processes, particularly in cells with high recombinational repair activity [90].

Circular single-stranded DNA (cssDNA) donors represent a promising alternative to linear ssODNs. Unlike linear ssDNA, cssDNA exhibits enhanced stability due to its closed circular structure, reducing exonuclease degradation in cells. cssDNA donors have been shown to improve HDR efficiency in multiple cell types, including iPSCs and primary T cells, by providing a persistent and highly accessible repair template. Their circular configuration also minimizes risks of off-target integration, making them a valuable tool for precise genome editing [91,92]. While ssODN and cssDNA templates hold great potential, their applications are limited to small-scale edits due to their restricted carrying capacity (Figure 2). Future research will likely explore methods to expand their versatility and optimize their delivery.

**Table 2:** Overview of donor DNA templates for HDR and their key features.

Template Type	Key Modifications	Suitable Applications	HDR Increase	Limitations
Viral Templates	Capsid engineering [67]	T cells, HSCs, and in vivo editing	Between ~3 and ~25-fold	Integration risk; immune response [70,71]
	Synthetic RNA-targeting sequences [69]			
ssODNs	Chemically modified (phosphorothioate) [82,83]	Point or small mutations; T cells, HEK293T, K562, and HSCs	~21% HDR	Limited capacity for large insertions
	Retron systems and transcription-coupled systems [85,86]		Between ~15% and ~60% HDR	
cssDNA	High stability; reduced degradation; minimizes off-target integration [91,92]	Precise small edits; iPSCs and T cells	Between ~20% and ~70% HDR	Limited capacity for large insertions
Plasmid Templates	Synthetic RNA-targeting sequences [93,94,95]	Large insertions in immortalized cell lines	Between ~10% and ~30% HDR	Cytotoxicity at high concentrations [96,97]
Linear dsDNA	TEG or RNA::DNA hybrids [98]	Large insertions with homology arms (200–800 bp), can be used in primary cells, γδ-T cells, and NK cells	~80% HDR	Cytotoxicity; random integration risks [96,97]
	Doggybone DNA [99]			
	Target sequences (tCTS) [100]		Between ~15% and ~30% HDR	
	Biotinylation [101]		~80%	

HDR efficiency reported in the table is context-dependent and varies based on donor template type, cell type, and delivery strategy. Viral vectors such as AAV demonstrate high HDR enhancement but pose risks of immune responses and genomic integration. Chemically modified ssODNs enable precise small edits, while cssDNA offers improved stability. Linear dsDNA with hybrid modifications or biotinylation can reach high HDR but may introduce cytotoxicity and random integrations. Plasmid-based repair templates support large insertions but show variable efficiency and potential toxicity at high concentrations.

#### 3.2.3. Double-Stranded DNA (dsDNA) Templates

Plasmid-based templates are widely utilized for HDR, particularly in immortalized cell lines, such as HEK293T and K562 cells. These templates are versatile due to their capacity to carry large DNA sequences and ease of construction. To enhance HDR efficiency, plasmids are often linearized near the homology arms to prevent concatemer formation and increase their availability for homologous recombination. Further advancements include incorporating synthetic RNA-targeting sequences into plasmids, enabling precise cleavage and an improved integration of the donor DNA [93,94,95]. While plasmid templates have shown limited effectiveness in primary cells like HSCs and T cells due to lower delivery efficiency and increased cytotoxicity, they remain a primary choice for research in robust immortalized systems [102,103,104].

Linear dsDNA templates, often derived from PCR products, are widely preferred for HDR in primary cells, including T cells, HSPCs, and iPSCs, where high precision and large insertions are required [105,106]. These templates are particularly effective for introducing transgenes and larger modifications. Strategies to enhance their efficiency include optimizing the homology arm length (400–800 bp) to balance HDR efficiency and minimize cytotoxicity [96,97]. End modifications, such as adding amine linkers (C6 and C12) [107], phosphorothioate (PS) linkages [108], and triethylene glycol (TEG) [98], significantly improve HDR by enhancing template stability, reducing concatemer formation, and directing repair toward homologous recombination. Further improvements have been achieved with doggybone DNA (dbDNA), an innovative closed-loop dsDNA format that prevents degradation, increases HDR efficiency, and has been successfully applied in CAR T cells and DNA vaccine production [99]. Additionally, strategies like truncated Cas9 target sequences (tCTS) incorporated into dsDNA templates enhance nuclear transport and HDR targeting, particularly in primary cells [93,100]. Modifications like biotinylation [101] or inert carbon spacers further suppress concatemer formation, reducing random integration and improving HDR fidelity [109]. Targeting cytosolic DNA sensors, such as the cGAS-STING pathway, is also being explored to mitigate immune responses and cytotoxicity caused by exogenous dsDNA [110]. Despite these advancements, challenges such as cytotoxicity at high concentrations and random integration events persist, necessitating continued optimization for therapeutic applications. Alongside DNA template modifications, protein engineering approaches, such as fusing Cas9 with DNA repair effectors, have gained significant popularity as a strategy to enhance HDR efficiency.

### 3.3. Enhancing HDR Using CRISPR-Cas9 Fusions Proteins

CRISPR–Cas9 fusion proteins offer a powerful strategy to tip the balance of HDR vs. NHEJ by modulating repair pathway choice, increasing donor template availability, and enhancing chromatin accessibility. These tailored approaches enable efficient and precise genome editing across a variety of cell lines and contexts (Table 3).

Fusions designed to bias repair pathway selection toward HDR are particularly effective in primary cells, where NHEJ activity is robust. For example, Cas9-CtIP, by promoting DNA end resection, facilitates the recruitment of Rad51 and other HDR factors, proving especially effective in fibroblasts and induced pluripotent stem cells (iPSCs) [111]. Likewise, Cas9-dnRNF168, which suppresses NHEJ by blocking 53BP1 recruitment, has demonstrated effectiveness across various cell lines [112]. Combining these functionalities, Cas9-CtIP-dnRNF168 exhibits a synergistic effect, significantly enhancing HDR efficiency and increasing the precision score by up to sevenfold in reporter cell lines. The reduction in NHEJ, coupled with enhanced HDR, also contributed to greater precision in primary cells, i.e., T cells and HSCs [112]. Cas9 fusion to dominant-negative dn53BP1 enhances HDR by inhibiting NHEJ specifically at cleavage sites [121]. Additionally, Cas9-RAD51 and Cas9-Brex27, targeting the homologous pairing and strand invasion steps of HDR, are particularly valuable for neuronal cells, where HDR efficiency is naturally low [113,114]. These fusions address the challenges associated with precise genome editing in non-dividing or slowly dividing cells. Moreover, Cas9-Geminin [120], which restricts Cas9 activity to the S and G2 phases, further enhances HDR by aligning editing events with the cell cycle’s HDR-permissive phases, making it an ideal candidate for HEK293T and iPSCs. The availability of donor templates is another critical determinant of HDR success. Fusions like Cas9-Avidin [115] and Cas9-SNAP [116], which tether biotinylated or covalently linked donor templates near the DSB, significantly enhance HDR efficiency in transiently transfected cells such as HEK293T and K562. In contrast, Cas9-Huh, which employs natural phospho-tyrosine bonding to attach single-stranded oligos (ssODNs), provides a scalable and cost-effective solution, achieving up to a 30-fold increase in HDR efficiency in HEK293T and U2OS cells [87]. These strategies are particularly suited for applications requiring the integration of large fragments or precision editing.

Chromatin architecture plays a pivotal role in determining the accessibility of repair machinery to the DSB. Condensed chromatin can hinder HDR, a limitation addressed by fusions like Cas9-HMGB1 and Cas9-HMGN1, which enhance chromatin accessibility and improve editing outcomes. In K562 and Jurkat cells, the dual-fusion Cas9-HMGB1-HMGN1 has demonstrated synergistic effects, achieving HDR increases of up to 3.4-fold compared to unmodified Cas9 [117,118]. Furthermore, epigenetic modifiers like Cas9-PRDM9, which catalyze histone methylation at the DSB, provide an additional layer of modulation, making them particularly effective for editing loci in heterochromatic regions or for applications requiring the precise control of chromatin states [119]. While fusion proteins enabled by modern protein engineering tools offer an innovative strategy to enhance HDR, especially at genomic loci or in cell types where HDR is suboptimal for clinical application, the approach still requires significant refinement. The large size of these fusion proteins can complicate proper folding and functional expression, and currently, few if any of these constructs are available as purified commercial proteins. Most applications rely on plasmid or mRNA delivery, which may be less effective than directly delivering recombinant protein. These limitations highlight the need for combinatorial strategies that integrate fusion protein approaches with other HDR-enhancing methods to optimize outcomes and improve the overall safety profile.

## 4. Emerging Strategies for Highly Precise Genome Editing

Highly robust, nuclease-based approaches also bring their own challenges such as toxicity due to DNA DSB, off-target effects, and inconsistent repair outcomes. These limitations underscore the need for alternative or complementary strategies [122]. Recently, several innovative technologies have gained traction by either avoiding DSBs altogether or bypassing reliance on the host cell’s DNA damage response. These emerging methods, broadly grouped into base editors, prime editors, recombinases, and RNA-guided transposon systems, offer the potential for more precise genetic modifications, improved safety profiles, and broader applicability across different cell types, including quiescent cells (Figure 2) [123].

Base editors (BEs) were the first widely adopted solution to circumvent the unintended outcomes of conventional CRISPR-based DSBs. By coupling a catalytically impaired Cas nuclease (dCas9 or a Cas9 nickase) to a DNA-modifying enzyme (e.g., cytidine deaminase or adenosine deaminase), BEs facilitate the direct conversion of one nucleotide base into another without requiring a donor template or homology-directed repair pathways [10]. Cytosine base editors (CBEs) [124] change C•G to T•A, whereas adenine base editors (ABEs) [125] convert A•T to G•C, collectively enabling the correction of the most common transition mutations that drive many genetic disorders. Ongoing refinements have enhanced base-editing precision by narrowing the editing window, improving sequence compatibility, and reducing off-target deamination [124,126,127,128,129]. While powerful for single-nucleotide variants and suitable for cells that do not readily divide, base editing cannot yet address large insertions, deletions, or transversions (e.g., C→G or A→T changes). Thus, BEs excel for diseases caused by relatively simple point mutations but are less amenable to complex or multi-site genome alterations.

Prime editing (PE) greatly expands on the concept of DSB-free editing by installing a reverse transcriptase (RT) domain onto a Cas9 nickase [130]. The “prime editor” uses a specialized guide RNA called a pegRNA (prime editing guide RNA), which not only directs Cas9 to the genomic target but also encodes the desired edit within its own sequence. The 3′ extension of the pegRNA serves as a template for new DNA synthesis by the RT domain, enabling single-base substitutions, small insertions, or deletions without introducing a full DSB or requiring an exogenous donor fragment [130]. Iterative versions of prime editors (PE2, PE3, and subsequent “PE max” variants) have improved on-target efficiency, reduced indels, and broadened the size of edits [131,132,133]. Although prime editing can theoretically install ~44 bp substitutions and up to ∼80 bp, it is typically less efficient when the desired insertion or deletion grows beyond a few dozen bases. Nevertheless, PE’s capacity for precise, template-based correction in both dividing and non-dividing cells, while minimizing DNA breaks, makes it a promising avenue for a wide range of genetic diseases.

Recombinases, originally discovered in phages and bacteria, catalyze the site-specific exchange of DNA segments flanked by recognition “attachment” sites. By repurposing enzymes like Bxb1, ϕC31, Cre, or Flp, researchers can integrate or excise substantial DNA segments in a predictable and highly site-specific manner. These systems do not rely on the cell’s endogenous DSB repair pathways, thus reducing the likelihood of unwanted indels [134,135,136]. Innovations such as “STRAIGHT-IN” (the serine and tyrosine recombinase-assisted integration of genes) demonstrate that large DNA payloads, up to and beyond 100 kb, can be integrated in human-induced pluripotent stem cells with high precision [137]. However, these methods often demand “landing pad” pre-installation (using CRISPR–Cas or other techniques) to provide recombinase attachment sites and typically require selection steps to enrich for the rare successful events [134]. Future efforts aim to streamline recombinase-driven approaches so they can be used in primary cells without laborious selection strategies, thereby broadening their clinical relevance.

Newly characterized Tn7-like transposons that utilize CRISPR-based targeting offer another exciting DSB-free editing strategy [138]. Here, a catalytically inactive Cas protein (sometimes in a Cascade complex) works with additional transposon-encoded factors to precisely insert large “cargo” DNA into predefined genomic locations. Because transposon insertion events do not require homologous recombination or the introduction of a DSB, they could provide a safer and potentially more efficient route for inserting large therapeutic sequences into non-dividing cells [139]. Early proof-of-concept studies in prokaryotes show high specificity and programmable integration, but achieving similarly robust performance in human cells remains a key challenge. Ongoing engineering aims to retool these multi-enzyme complexes for efficient delivery and stable insertion in mammalian systems, raising the possibility of harnessing CRISPR-guided transposases for future gene therapies that require large genomic modifications.

## 5. Conclusions

As the field of genome editing continues to expand, homology-directed repair (HDR) remains a critical cornerstone. HDR is uniquely suited to introducing substantial or precise changes into the genome, making it especially advantageous for diseases with multiple mutational hotspots or for larger-scale insertions and replacements. While more recent strategies, such as base and prime editors, elegantly bypass the complexities of double-strand break repair and excel at smaller edits, recombinases and CRISPR-guided transposons open the door to larger gene insertions without relying on host cell DNA repair. Yet no one can fully recapitulate the breadth of edits that HDR allows; each platform has its own strength and weaknesses. The enduring appeal of HDR lies in its versatility: with the fine-tuning of donor design, cell-cycle regulation, and DNA repair factor modulation, it can address a broad spectrum of genetic pathologies.

The future of HDR-based genome editing lies in enhancing its precision and reliability for clinical applications. One of the major challenges ahead is the efficient and safe delivery of both the Cas protein and the repair template. To address this, smaller Cas variants are being explored for compatibility with viral vectors, enabling more efficient in vivo expression. At the same time, lipid nanoparticles are gaining attention as a promising platform for the targeted delivery of donor DNA. The rise of artificial intelligence (AI) is also transforming the field of protein engineering. AI can now be used to design peptides or predict protein fusions that enhance HDR outcomes. Moreover, machine learning models are being developed to optimize editing conditions by predicting the ideal combination of small molecules, repair templates, and fusion proteins for specific genomic loci. A modular and combinatorial approach, tailored to the biological context, will likely yield the safest and most effective strategies for precise genome editing.

## Figures and Tables

**Table 3:** Cas9 fusion proteins for enhancing HDR and their reported fold increase.

Category	Fusion Protein	Mechanism	Cell Lines	HDR Increase
Repair Pathway Modulation	Cas9-CtIP	Promotes DNA resection	Fibroblasts and iPSCs	Between ~1- and ~2-fold [111]
	Cas9-dnRNF168	Blocks NHEJ by inhibiting 53BP1 recruitment	HSPCs and T cells	Not specified [112]
	Cas9-CtIP-dnRNF168	Synergistically promotes HDR and inhibits NHEJ	K562, Jurkat, and HSPCs	Up to 7-fold [112]
	Cas9-RAD51	Promotes strand invasion	Multiple cell lines	~1-fold [113]
	Cas9-Brex27	BRCA2 domain recruiting Rad51	iPSCs and fibroblasts	Between ~2- and ~3-fold [114]
Repair Template Saturation	Cas9-Avidin	Binds biotinylated templates	HEK293T and K562	~2-fold [115]
	Cas9-SNAP	Covalently attaches donor DNA	HEK293T and K562	Between ~3- and ~24-fold [116]
	Cas9-Huh	Tethers ssDNA donors via phospho-tyrosine bonds	HEK293T and U2OS	Between ~15- and ~30-fold [87]
Chromatin Modulation	Cas9-HMGB1	Increases chromatin accessibility	K562 and Jurkat	Between ~2- and ~3-fold [117,118]
	Cas9-HMGN1	Increases chromatin accessibility	K562 and Jurkat	~2-fold [117,118]
	Cas9-HMGB1-HMGN1	Dual fusion for synergistic chromatin effects	K562 and Jurkat	~3-fold [117,118]
	Cas9-PRDM9	Histone methyltransferase-promoting HDR	HEK293T	~2-fold [119]
Cell Cycle Control	Cas9-Geminin	Restricts Cas9 activity to S/G2 phases	HEK293T and iPSCs	~2-fold [120]

Summarizes various Cas9 fusion strategies categorized by their mechanism of HDR enhancement. Fold increases in HDR efficiency are based on gene knock-in studies and may vary depending on target locus, delivery method, and donor type. Most results are derived from human cell lines such as HEK293T, K562, Jurkat, and iPSCs.

## Data Availability

No new data were created or analyzed in this study. Data sharing is not applicable to this article.
